# RNA demethylase ALKBH5 promotes colorectal cancer progression by posttranscriptional activation of RAB5A in an m6A‐YTHDF2‐dependent manner

**DOI:** 10.1002/ctm2.1279

**Published:** 2023-05-18

**Authors:** Dingcheng Shen, Jinxin Lin, Yumo Xie, Zhuokai Zhuang, Gaopo Xu, Shaoyong Peng, Guannan Tang, Liangliang Bai, Mingxuan Zhu, Yu Zhang, Ziying Huang, Puning Wang, Xiaoxia Liu, Meijin Huang, Yanxin Luo, Xiaolin Wang, Huichuan Yu

**Affiliations:** ^1^ Department of Colorectal Surgery The Sixth Affiliated Hospital Sun Yat‐sen University Guangzhou Guangdong China; ^2^ Guangdong Institute of Gastroenterology Guangzhou Guangdong China; ^3^ Department of General Surgery The Sixth Affiliated Hospital Sun Yat‐sen University Guangzhou Guangdong China; ^4^ Guangdong Provincial Key Laboratory of Colorectal and Pelvic Floor Diseases The Sixth Affiliated Hospital Sun Yat‐sen University Guangzhou Guangdong China; ^5^ Zhongshan School of Medicine Sun Yat‐sen University Guangzhou Guangdong China

**Keywords:** ALKBH5, colorectal cancer (CRC), N6‐methyladenosine (m6A), RAB5A

## Abstract

**Background:**

N6‐methyladenosine (m6A) modification is an emerging epigenetic regulatory mechanism in tumourigenesis. Considering that AlkB homolog 5 (ALKBH5) is a well‐described m6A demethylase in previous enzyme assays, we aimed to investigate the role of m6A methylation alteration conferred by disturbed ALKBH5 in colorectal cancer (CRC) development.

**Methods:**

Expression of ALKBH5 and its correlation with clinicopathological characteristics of CRC were evaluated using the prospectively maintained institutional database. The molecular role and underlying mechanism of ALKBH5 in CRC were explored using in vitro and in vivo experiments with methylated RNA immunoprecipitation sequencing (MeRIP‐seq), RNA‐seq, MeRIP‐qPCR, RIP‐qPCR and luciferase reporter assays.

**Results:**

ALKBH5 expression was significantly upregulated in CRC tissues compared to the paired adjacent normal tissues, and higher expression of ALKBH5 was independently associated with worse overall survival in CRC patients. Functionally, ALKBH5 promoted the proliferative, migrative and invasive abilities of CRC cells in vitro and enhanced subcutaneous tumour growth in vivo. Mechanistically, RAB5A was identified as the downstream target of ALKBH5 in CRC development, and ALKBH5 posttranscriptionally activated RAB5A by m6A demethylation, which impeded the YTHDF2‐mediated degradation of RAB5A mRNA. In addition, we demonstrated that dysregulation of the ALKBH5‐RAB5A axis could affect the tumourigenicity of CRC.

**Conclusions:**

ALKBH5 facilitates the progression of CRC by augmenting the expression of RAB5A via an m6A‐YTHDF2‐dependent manner. Our findings suggested that ALKBH5‐RAB5A axis might serve as valuable biomarkers and effective therapeutic targets for CRC.

## Graphical abstract

ALKBH5 was significantly upregulated in CRC tissues and could facilitate the proliferation and motility of CRC cells. RAB5A, a member of RAS oncogene family, was a direct target of ALKBH5 and served as a novel oncoprotein in CRC. ALKBH5 facilitates the progression of CRC by augmenting the expression of RAB5A via an m6A‐YTHDF2‐dependent manner.

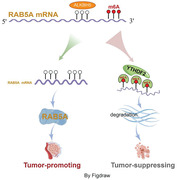

## 1 INTRODUCTION

Colorectal cancer (CRC) is one of the most prevailing malignancies worldwide and also the third‐leading cause of cancer‐related mortality despite tremendous progress witnessed in CRC prevention, diagnosis and treatment during past decades.^1^, ^2^, ^3^ Viewing this, it is imperative to further explore the molecular mechanisms underlying CRC tumourigenicity with the aim of developing more promising diagnostic and prognostic biomarkers as well as effective therapeutic strategies.

As the predominant kind of RNA modification in eukaryotes, N6‐methyladenosine (m6A) is now attracting increasing attention since it extensively affects various biological processes associated with human diseases, especially with cancers.^4^, ^5^ The process of m6A modification is dynamically and reversibly regulated by several writers (methyltransferases), erasers (demethylases) and readers (effector proteins), which account for mRNA metabolism including translation, splicing, transportation, stability and degradation.^6^, ^7^ Actually, some recent studies have demonstrated that these m6A regulatory proteins were widely participated in the malignancy of CRC.^8^, ^9^ For example, as the m6A writers, methyltransferase‐like 3 (METTL3) has been identified to promote CRC progression by maintaining the expression of SOX2 via an m6A‐IGF2BP2‐dependent manner,^10^ while METTL14 acts as a tumour suppressor and promotes lncRNA XIST and SOX4 mRNA methylation to attenuate their expression through YTHDF2‐mediated RNA decay.^11^, ^12^ Moreover, as the first defined m6A erasers, fat‐mass and obesity‐associated protein (FTO) could increase MYC expression by preventing its m6A modification and further promote the occurrence and progression of CRC.^13^ However, as a well‐described m6A demethylase, although AlkB homolog 5 (ALKBH5) was reported to play vital roles in multiple cancers, such as pancreatic cancer,^14^, ^15^ intrahepatic cholangiocarcinoma,^16^ glioblastoma^17^ and osteosarcoma,^18^, ^19^ few studies have ever extensively explored the role of ALKBH5 in CRC.

In the present study, we comprehensively explored the carcinogenic mechanisms of ALKBH5 in CRC from clinicopathological characteristics to cellular mechanisms. We demonstrated that ALKBH5 was upregulated in CRC, and higher expression of ALKBH5 indicated an adverse prognosis. ALKBH5‐induced m6A demethylation significantly promoted the proliferation and invasiveness of CRC cells through m6A‐YTHDF2‐dependent posttranscriptional activation of RAB5A.

## 2 MATERIALS AND METHODS

### 2.1 Patients and samples

Paired tumour and adjacent normal tissues from 35 CRC patients after curative‐intent resection were collected at the Sixth Affiliated Hospital of Sun Yat‐sen University (SAH‐SYSU). To determine the correlation between ALKBH5 expression and clinic‐pathological characteristics of CRC, a total of 201 CRC patients combined with their fresh frozen tumour specimens originating from the institutional database program of colorectal disease (IDPCD) at our institute were also included in this study. The IDPCD cohort integrated patient data, including demographic and clinic‐pathological characteristics and follow‐up data, which was described in previous publications.^20^, ^21^, ^22^

### 2.2 Cell culture and transfection

All cell lines were obtained from the American Type Culture Collection (ATCC), and cultured in ATCC‐recommended media (Gibco, Grand Island, NY, USA) supplementing with FBS (Gibco; 10%, v/v) and 1% penicillin‐streptomycin at a 37°C incubator with 5% CO_2_. The cells also routinely underwent PCR evaluation for *Mycoplasma*‐free using a *Mycoplasma* detection kit (TaKaRa). The ALKBH5 plasmid, shALKBH5 plasmid, shRAB5A plasmid, and their control plasmids as well as siYTHDF1/2/3 were purchased from TSINGKE (Tsingke Biotechnology Co., Ltd. China). The constructs were confirmed by direct sequencing in an independent third party (Sangon) before use. HCT116 and SW480 cells were transfected using corresponding plasmids with the reference to manufacturer's instructions, and qPCR and western blotting were applied to assess transfection efficiency. Details of the target sequences used in this study were listed in Table S1.

### 2.3 RNA extraction and quantitative real‐time PCR

Genomic RNA was extracted from tissues and CRC cells using RNeasy Mini Kit (Qiagen, Germany) following its recommended protocols. Then RNA was reverse transcripted into cDNA according to the recommended instructions (TaKaRa, Japan). PCR using custom‐designed primers and cDNA templates was conducted using an SYBR Green Mix system (Applied Biosystems, USA), and detailed methods and data analysis were reported in our previous study.^23^ The primers used are listed in Table S2.

### 2.4 Protein isolation and western blotting

The proteins isolated from cell lysates were then electrophoresed by SDS‐PAGE and immunoblotted following classic protocols as we described previously.^24^, ^25^ The primary antibodies were used as follows: anti‐ALKBH5 (Abcam, ab195377), anti‐RAB5A (Abcam, ab218624), anti‐YTHDF2 (Proteintech, 24744‐1‐AP) and anti‐GAPDH (Proteintech, 10494‐1‐AP).

### 2.5 Immunohistochemistry (IHC)

All tissues were embedded in paraffin wax and sectioned into 3 µm slices. Standard protocols of IHC have been described previously.^26^ The deparaffinised sections were incubated with anti‐ALKBH5 (Abcam, ab195377), anti‐RAB5A (Abcam, ab218624), and anti‐Ki67 (Cell Signaling Technology, 9449).

### 2.6 Immunofluorescence (IF)

A total of 2 × 10^5^ cells were cultured in a glass‐bottom cell culture dish for 24 h and then fixed with paraformaldehyde, and the membrane was permeated with Triton X‐100, followed by blocking using goat serum (Thermo Fisher Scientific). RAB5A antibody (Abcam, ab218624) and fluorescent secondary antibody were used to develop the expression of RAB5A. The nuclei of the cells were stained with DAPI. The fluorescence was photographed by a Leica TCS SP8 microscope.

### 2.7 In vitro cell proliferation, migration and invasion assays

Cell proliferation was assessed by Cell Counting Kit‐8 (APExBIO, USA) (3000 cells/well were seeded in triplicate at a 96‐well plate) and colony formation (1000 cells/well were seeded at a 6‐well plate). Tanswell chamber assays (1 × 10^5^ cells in 200 µL serum‐free media were seeded in the top chamber) were applied to evaluate cell migrative and invasive abilities. Standard procedures have been described previously.^27^

### 2.8 Tumour xenograft experiments

All animal experiments were approved by the animal care Committee of Sixth Affiliated Hospital of Sun Yat‐sen University (No. IACUC‐2022022401). BALB/c‐nude mice (4−6 weeks old) were obtained from the Vital River. Briefly, 4 × 10^6^ transfected HCT116 cells or 8 × 10^6^ transfected SW480 cells in 0.1 mL PBS were injected into mice subcutaneously, and these mice were randomly divided into the experimental and control group. The tumours were monitored, calculated, removed and weighed as well as further fixed and embedded following corresponding protocols as described previously.^27^

### 2.9 m6A dot‐blot assay

Total RNA was isolated from CRC cells using RNeasy Mini Kit (Qiagen, Germany) following its recommended protocols. Then RNAs were serially diluted to 50, 100 and 200 ng/µL using RNase free water, and 100, 200 and 400 ng RNAs were spotted to dot‐blot assays as introduced by previous publication.^28^ The primary antibody used was anti‐m6A (Abcam, ab151230).

### 2.10 RNA sequencing, methylated RNA immunoprecipitation and m6A sequencing (MeRIP‐seq), and data analysis

Total RNA was collected from HCT116 cells with stably ALKBH5‐knockdown and control cells (two replications, which were labelled as shALKBH5 and shNC, respectively) with a TRIzol reagent (Invitrogen, CA). RNA‐seq was conducted using Illumina NovaSeq 6000 platform. Paired‐end clean reads were aligned to the human reference genome (Ensemble_GRCh38.104). Differential expression analysis was performed using DESeq2 R package (1.20.0). For m6A‐RIP and MeRIP‐seq, chemically fragmented RNAs were incubated with anti‐m6A antibody for immunoprecipitation according to the standard procedures of the Magna MeRIP™ m6A Kit (Millipore, Germany). Enrichment of the m6A‐containing mRNA was then analysed either through quantitative reverse‐transcription polymerase chain reaction (MeRIP‐qPCR) or by high‐throughput sequencing. Enrichment of m6A mRNAs was then used for library construction with the NEBNext Ultra RNA library Prep kit from Illumina (NEB) and analysed via high‐throughput sequencing using Illumina Hiseq X platform. RNA‐seq, MeRIP‐seq and the sequencing data analysis were largely supported by Novogene (Beijing, China).

### 2.11 RNA immunoprecipitation (RIP)

The RIP assay was conducted using the RIP kit (BersinBio, China) according to the manufacturer's guidelines. Briefly, magnetic beads were first incubated with 5 µg of anti‐ALKBH5 (Millipore, ABE547), YTHDF2 (Proteintech, 24744‐1‐AP) and normal IgG (Beyotime, A7016) at 4°C overnight. After the incubation in digestion buffer containing proteinase K, RNAs of interest were eluted from RNA‐protein complexes and then purified for further validation by qPCR to assess the enrichment.

### 2.12 RNA stability assays

Seeding stably transfected CRC cells into a 6‐well plate for 24 h and then treating them with actinomycin D (Sigma‐Aldrich, HY‐17559) at 5 µg/mL for 0, 3, and 6 h, followed by RNA extraction. The remaining RAB5A mRNA was measured by qPCR as described above.

### 2.13 Luciferase reporter assay

According to the MeRIP‐seq results, the sites of RAB5A transcript where differential enrichment of m6A motif were focused to conduct luciferase reporters containing either wild‐type or mutant (N6‐methylated adenosines were replaced by cytosines) RAB5A 3′UTR sequences into the pmirGLO Basic vector by TSINGKE Biological Technology (Beijing, China). Detailed descriptions of the wild‐type and m6A sites mutated regions of RAB5A were shown in Table S3. Subsequently, CRC cells were transfected with the constructed luciferase reporter plasmids after being cultivated for 48 h. The luciferase activity was measured through a Firefly & Renilla Luciferase Reporter Assay Kit (Meilunbio, China) following the recommended instructions.

### 2.14 Statistical analysis

The statistical analysis of the differences among the intergroup comparisons were based on a two‐tailed Student's *t* test or one‐way analysis of variance (ANOVA). The Wilcoxon group signed‐rank test was adopted to compare two group samples, which were not normally distributed. Plot survival curves were plotted by Kaplan–Meier analysis, and the statistical significances were compared through a log‐rank test. A chi‐square test was applied to analyse qualitative data. Univariate and multivariate analysis based on the Cox hazards regression were conducted to estimate the prognostic factors correlated with the clinical variables. Statistical correlation analysis of the expression of ALKBH5 and RAB5A was conducted with linear regression. The statistical analyses were performed through GraphPad Prism 8.0 (Inc., USA), SPSS 22.0 (Inc., USA) and R‐project 3.5.1. Experiments were performed and repeated at least three times independently. *p < .05, **p < .01 and ***p < .001 indicated statistically significant, and ‘ns’ was considered with no statistical significance (*p* > .05).

## 3 RESULTS

### 3.1 ALKBH5 is overexpressed in CRC and serves as an unfavourable prognostic factor for CRC

**FIGURE 1:**
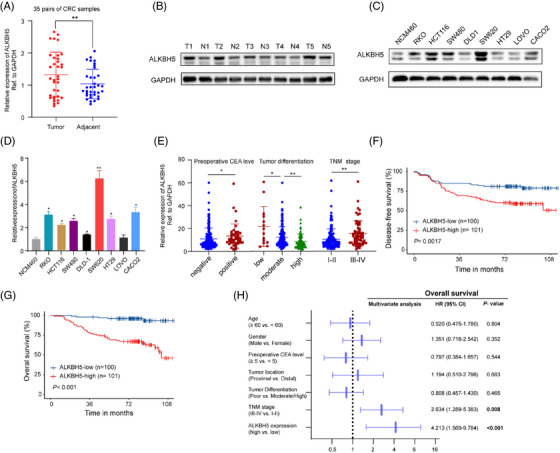
ALKBH5 is highly expressed and associated with adverse survival in patients with CRC. (A) qPCR was used to analyse the mRNA expression of ALKBH5 in 35 paired tumour and adjacent normal tissues. (B) Five pairs of CRC samples were subjected to western blotting analysis of ALKBH5. T: tumour; N: adjacent normal tissue. (C, D) The expression of ALKBH5 in different CRC cell lines compared with NCM460 detected using western blotting (C) and qPCR (D). (E) The association between the mRNA expression of ALKBH5 and preoperative CEA level, tumour differentiation and TNM stage among 201 CRC patients (negative, CEA < 5 ng/mL; positive, CEA ≥ 5 ng/mL). (F, G) Kaplan–Meier analysis of disease‐free survival (DFS, F) and overall survival (OS, G) among CRC patients based on ALKBH5 mRNA expression in tumour tissues. The log‐rank test was used to analyse the significance of the differences between groups. (H) Multivariate analysis was employed for CRC patients using COX regression model based on those clinicopathologic variables. Symbols and bars in forest plots correspond to hazard ratio (HR) and 95% confidence interval (CI), respectively. *p < .05, **p < .01, ***p < .001.

To explore the expression of ALKBH5 in CRC, we first detected the levels of mRNA and protein in fresh frozen tumours and matched adjacent normal tissues using qPCR, WB and IHC assays, respectively. These results revealed that ALKBH5 was upregulated significantly in CRCs (Figures 1A and B and S1). Then, we also examined ALKBH5 levels in CRC cell lines, ALKBH5 was obviously overexpressed in most CRC cells compared to normal mucosal cell lines (Figure 1C and D). To further investigate the relevance of ALKBH5 expression in CRC clinically, a correlation analysis were conducted among clinicopathologic variables from an in‐house cohort consisting of 201 pathologically confirmed CRC patients and corresponding ALKBH5 expression of fresh frozen tumour tissues by qPCR assays. A high level of ALKBH5 was significantly associated with positive preoperative CEA levels (*p* = .010), more aggressive differentiation (*p* = .013) and advanced TNM stages (*p* = .001) (Figure 1E and Table 1). The Kaplan–Meier survival analysis showed that CRC patients characterised with higher ALKBH5 had extremely worse disease‐free and overall survival (DFS and OS) (Figure 1F and G). In addition, multivariate regression analysis exhibited that overexpressed ALKBH5 was demonstrated as an independent prognostic factor in CRC patients (HR = 4.213, *p* < .001) (Figure 1H). Collectively, these results implied that ALKBH5 is upregulated as well as has significant clinical relevance in CRC and acted as a prognostic biomarker for CRC patients.

**TABLE 1:** Correlation analysis between clinicopathologic variables and ALKBH5 expression in CRC patients

		ALKBH5 expression		
Characteristics	*n* = 201	Low (*n* = 100)	High (*n* = 101)	*p* Value
**Age (years)**				.838
< 60	87 (43.2%)	44 (44.0%)	43 (42.6%)	
≥ 60	114 (56.8%)	56 (56.0%)	58 (57.4%)	
**Gender (*n*)**				.099
Male	115 (57.2%)	63 (63.0%)	52 (51.5%)	
Female	86 (42.8%)	37 (37.0%)	49 (48.5%)	
**Preoperative CEA level (ng/mL)**				**.010**
< 5	160 (79.6%)	87 (87.0%)	73 (72.3%)	
≥ 5	41 (20.4%)	13 (13.0%)	28 (27.7%)	
**Tumour location (*n*)**				.882
Proximal	37 (18.4%)	18 (18.0%)	19 (18.8%)	
Distal	164 (81.6%)	82 (82.0%)	82 (81.2%)	
**Tumour Differentiation (*n*)**				**.013**
High	68 (33.8%)	43 (43.0%)	25 (24.8%)	
Moderate	119 (59.2%)	53 (53.0%)	66 (65.3%)	
Poor	14 (7.0%)	4 (4.0%)	10 (9.9%)	
**TNM stage (*n*)**				**.001**
I‐II	142 (70.6%)	81 (81.0%)	61 (60.4%)	
III‐IV	59 (29.4%)	19 (19.0%)	40 (39.6%)	

Cutoffs for grouping were determined by the median of ALKBH5 expression in the cohort.

### 3.2 ALKBH5 promotes CRC cell growth and motility

**FIGURE 2:**
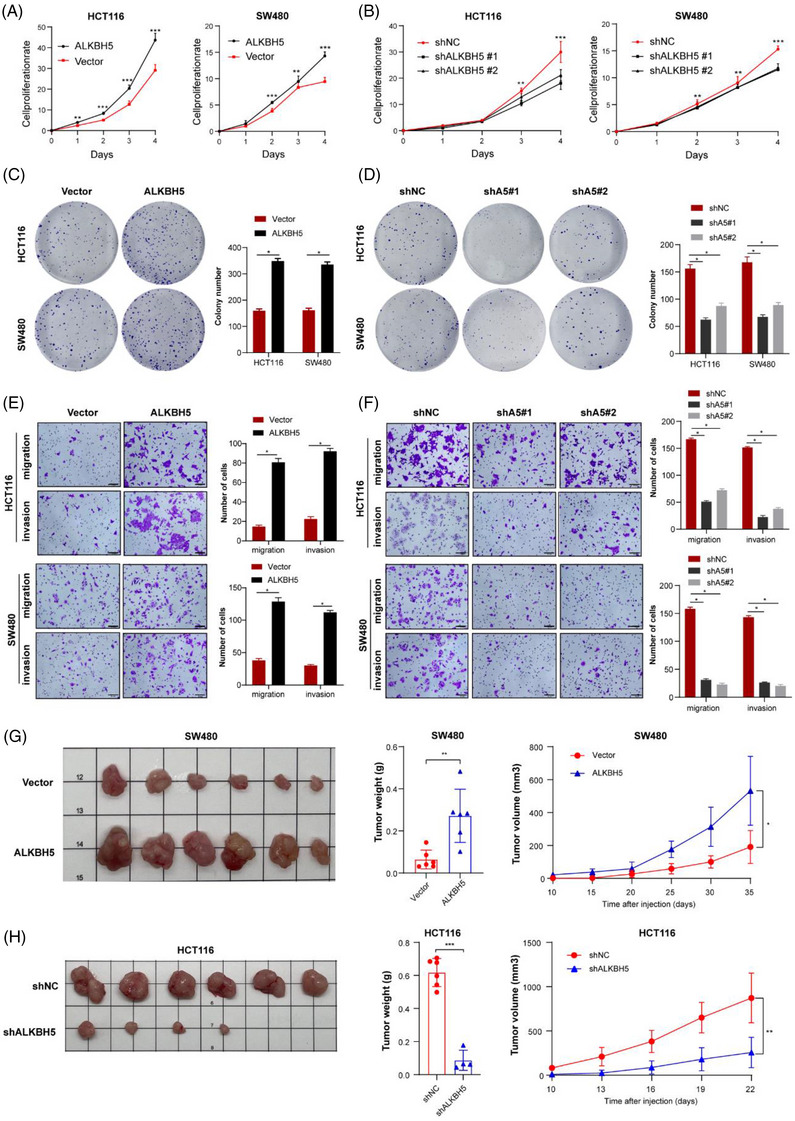
ALKBH5 promotes the growth and motility of CRC. A‐D CCK‐8 (A, B) and colony formation (C, D) assays were applied to evaluate the proliferation ability of HCT116 and SW480 cells with knockdown or overexpression of ALKBH5. (E, F) Transwell assays of HCT116 and SW480 cells were applied to measure their migration and invasion abilities (scale bars, 50 µm). (G, H) Representative subcutaneous xenograft tumour image from sacrificed mice and their tumour weight and tumour volume curves were shown with models injected with stable ALKBH5‐overexpressing SW480 cells (*n* = 6, G) or ALKBH5‐knockdown HCT116 cells (*n* = 6, H), the remaining two mice had no tumours in the ALKBH5‐knockdown group. (Scale plate: 10 mm x 10 mm for each square). Histograms besides presented the numbers of each group.

To evaluate the function of ALKBH5 in CRC, ALKBH5 was silenced through two shRNA targeting the ALKBH5 (shA5#1, shA5#2), while stably upregulated through a lentivirus vector in HCT116 and SW480 cells according to the ALKBH5 expression in CRC cells (Figure 1C and D). The transfection efficiency was validated by WB and qPCR (Figure S2). CCK‐8 assays and colony formation indicated that the up‐regulation of ALKBH5 enhanced the proliferative ability of CRC cells (Figure 2A and C), while knockdown of ALKBH5 showed the opposite effect (Figure 2B and D). The Transwell assays suggested that the overexpression of ALKBH5 apparently increased both migration and invasion of CRC (Figure 2E), while attenuation of the expression significantly impaired those phenotypes (Figure 2F). To further examine the carcinogenic effect of ALKBH5 in CRC, we performed the in vivo experiments with subcutaneous xenograft. We found that the volumes, weights and growth rate of xenografted tumours significantly increased compared to control group when ALKBH5‐overexpressed cells were implanted (Figure 2G). On the contrary, silencing ALKBH5 expression drastically inhibited tumour weight and growth (Figure 2H). Moreover, the expression of Ki‐67 was also tested along with the diverse ALKBH5 expression (Figure S3). On the whole, these results revealed that ALKBH5 serves as an oncogene to promote CRC malignancy.

### 3.3 RAB5A is a downstream target of ALKBH5

**FIGURE 3:**
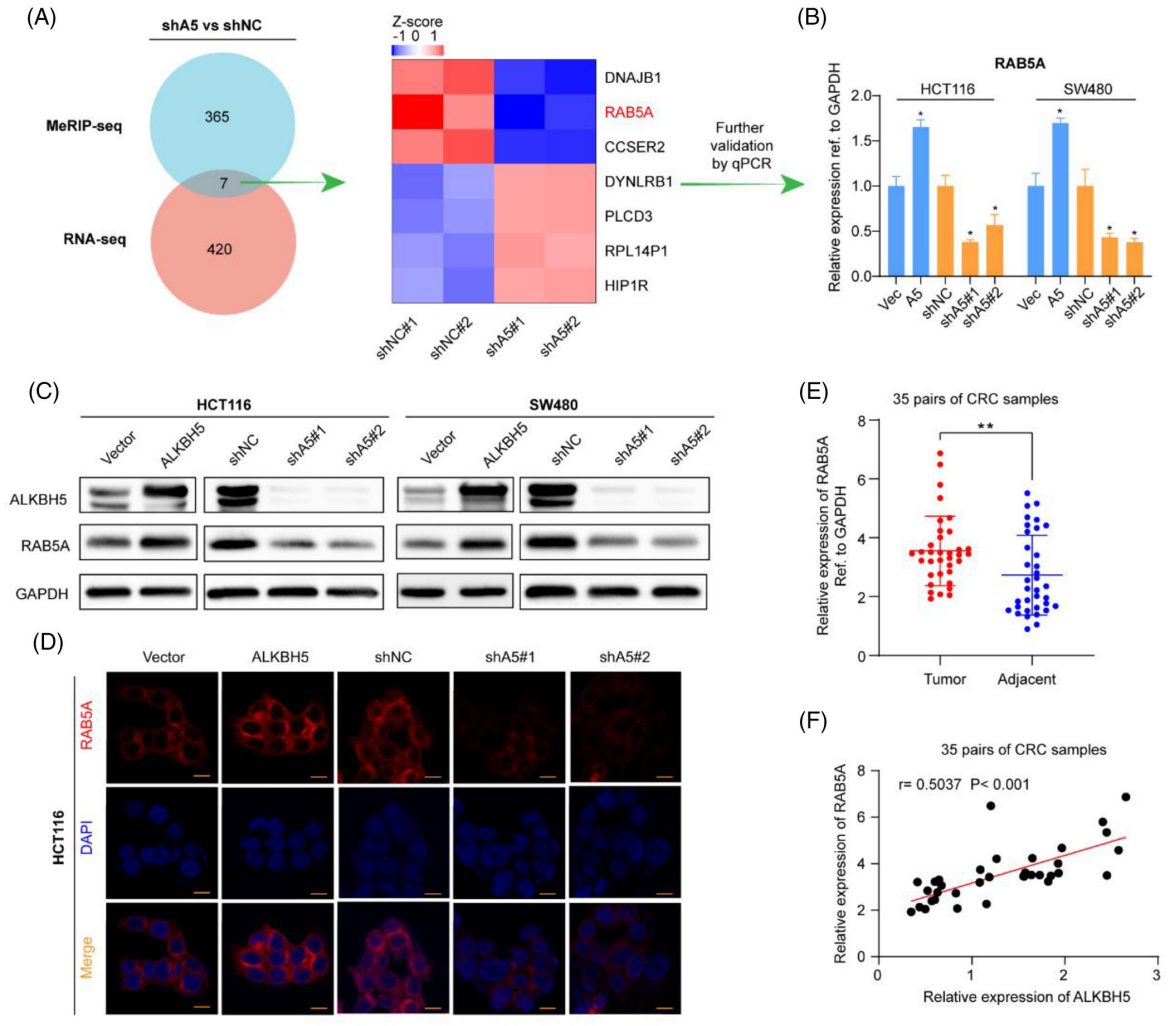
RAB5A is identified as a downstream target of ALKBH5. (A) MeRIP‐Seq and RNA‐Seq identified differentially expressed genes in HCT116 stable knockdown cells when compared with their corresponding controls using the Venn diagram, and the 7 overlapped differentially expressed genes showed in the heat map were subject to following validation using qPCR. (B–D) RAB5A was examined in ALKBH5‐silenced or ‐overexpressing cells through qPCR (B), western blotting (C) and immunofluorescence (D) (scale bars, 50 µm), respectively. (E) qPCR was used to analyse the mRNA expression of RAB5A in 35 paired tumour and adjacent normal tissues. (F) Correlation analysis of ALKBH5 expression and RAB5A expression in CRC tissues.

In order to further investigate the precise mechanism of ALKBH5 in the malignancy of CRC, we employed an integrated approach by combining RNA‐seq and MeRIP‐seq using stable ALKBH5‐knockdown HCT116 cells and the corresponding control cells. RNA‐seq data revealed that 427 differential transcripts were significantly regulated upon ALKBH5‐knockdown. We calculated the fold change of gene expression and considered genes with |log_2_FC| > 0.5 and *p*adj < .05 as statistically different expression (Supplementary file 2: RNA‐seq data). Meanwhile, MeRIP‐seq revealed that the m6A peaks of 372 transcripts showed increased enrichment upon ALKBH5 silence (log_2_FC > 0 and *p*adj < 0.05) (Supplementary file 2: MeRIP‐seq data). In this study, Venn analysis was performed by combining with the differentially expressed genes identified via RNA‐seq and RIP‐seq (Figure 3A), and analysis showed that seven genes were overlapped, namely, DNAJB1, RAB5A, CCSER2, DYNLRB1, PLCD3, RPL14P1 (pseudogene) and HIP1R. Then, they were scheduled for initial validation in ALKBH5‐ overexpressing or ‐silencing cells through qPCR. Intriguingly, only RAB5A was the only gene, which was consistently identified to be positively mediated by ALKBH5 in all cells (Figures 3B and S4). Moreover, as indicated by western blotting and immunofluorescence, the levels of RAB5A were increased in ALKBH5‐overexpressing cells and decreased in ALKBH5‐knockdown cells (Figures 3C and D and S5), which were further confirmed by IHC assay using xenograft tissues in nude mice (Figure S3). Furthermore, the levels of RAB5A mRNA were also increased in tumour tissues (Figure 3E) and showed a positive correlation with those of ALKBH5 in CRC tissues (Figure 3F). As a result, a preliminary conclusion could be drawn that RAB5A might be the direct downstream target of ALKBH5.

### 3.4 ALKBH5 knockdown abolishes RAB5A mRNA stability through an m6A‐YTHDF2‐dependent mechanism

**FIGURE 4:**
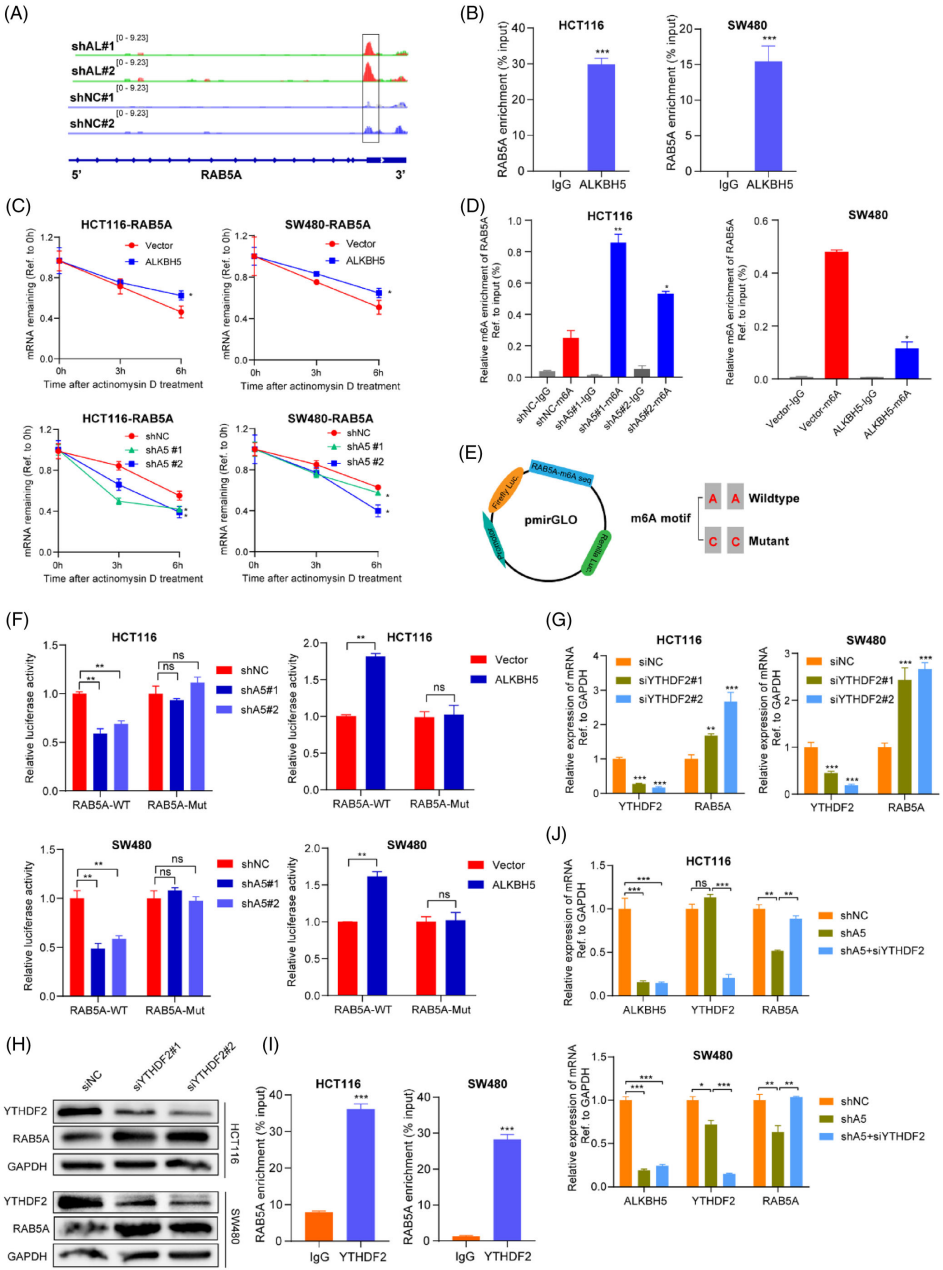
ALKBH5 enhances the stability of RAB5A mRNA via an YTHDF2‐m6A‐dependent pattern. (A) m6A abundance on RAB5A mRNA in negative control or ALKBH5‐knockdown HCT116 cells was plotted by the IGV. Green and grey colours show the m6A signals of input samples, while red and blue stand for signals of IP samples. At the same position, m6A peaks of IP group over input group were recognised as the genuine m6A level. (B) RIP‐qPCR assay for the enrichment of RAB5A mRNA on anti‐ALKBH5 antibody or IgG negative control antibody in HCT116 and SW480 cells. (C) The expression levels of Rab5A in different transfected and control cells were quantified by qPCR at indicated time points after actinomycin D treatment. (D) MeRIP‐qPCR analysis of Rab5A m6A modification levels in HCT116 cells transfected with knockdown of ALKBH5 or SW480 cells transfected with overexpression of ALKBH5 and their corresponding controls. (E) Graphical explanation for construction of luciferase reporters. The wild‐type or mutant (N6‐methylated adenosines were replaced by cytosines among m6A motif) sequence of RAB5A−3′UTR was inserted into a pmirGLO vector between Firefly and Renilla elements. (F) The dual luciferase reporter gene assay was conducted to detect the luciferase activity among cells transfected with the RAB5A‐wild‐type or ‐mutated plasmids. (G, H) YTHDF2 was knockdown in two CRC cells followed by the measurement of RAB5A expression via qPCR (G) and western blotting (H). (I) RIP‐qPCR assay for the enrichment of RAB5A mRNA on anti‐YTHDF2 antibody or IgG negative control antibody in HCT116 and SW480 cells. (J) Rescue assays were applied to verify the impact of YTHDF2 on ALKBH5‐mediated modulation of RAB5A.

To further verify the direct mechanism that ALKBH5 targets RAB5A mRNA and whether it depends on the m6A catalytic activity. We first detected the global m6A levels in different transfected CRC cells and their control groups through m6A dot‐blot assays. As expected, the m6A levels of total RNA were decreased obviously in both ALKBH5‐overexpression HCT116 and SW480 cells compared with vector cells, while ALKBH5‐deletion had the opposite effects (Figure S6). The MeRIP‐seq analysis showed that m6A‐peak of RAB5A in 3′UTR accreted apparently with the knockdown of ALKBH5 (Figure 4A). RIP assays performed by the anti‐ALKBH5 antibody in HCT116 and SW480 cells validated that RAB5A mRNA was enriched significantly in the ALKBH5 group, but not the IgG group (Figure 4B). Next, we treated cells with actinomycin D to block transcription and assess the effect of ALKBH5 on RAB5A mRNA stability. We found that ALKBH5 sufficiency enhanced the stability of RAB5A, whereas ALKBH5 knockdown induced a faster degradation rate of RAB5A mRNA (Figure 4C). The above results demonstrated that ALKBH5 was bound to RAB5A and increased the stability of RAB5A mRNA via a posttranscriptional mechanism.

Then MeRIP‐qPCR with specific primers was performed to detect the enrichment of m6A in RAB5A. The results demonstrated that the m6A modification of RAB5A mRNA was significantly elevated on ALKBH5 knockdown as well as substantially reduced on activation of ALKBH5 (Figure 4D). To clarify the essential role of m6A modification on RAB5A, luciferase reporters containing either wild‐type or mutant (N6‐methylated adenosines were replaced by cytosines) RAB5A 3′UTR sequences were designed (Figure 4E). The luciferase activities of CRC cells transfected with RAB5A‐WT plasmid, but not the mutant counterpart, obviously decreased in the silence of ALKBH5, and the analogous results would be seen in ALKBH5‐overexpressed cells (Figure 4F), which revealed that the expression of RAB5A mRNA was under the regulation of ALKBH5‐related m6A modification.

It was acknowledged that m6A ‘readers’ were crucially involved in recognising methylated target transcripts; we next planned to investigate the potential reader protein participating in the procedure elucidated above. Given that YTHDFs were reported to extensively mediate translation efficiency and degradation of m6A modified RNAs,^29^ we hence explored the effect of YTHDF1/2/3 on the stability RAB5A mRNA. Two specific designed siRNAs targeting YTHDF1/2/3 were used in two CRC cells to validated the alterations of RAB5A expression. We found that YTHDF2 knockdown remarkably augmented RAB5A expression (Figure 4G and H), but YTHDF1/3 exhibited no effect on RAB5A in two cells (Figure S7). Moreover, YTHDF2 deficiency could enhance the stability of RAB5A in two CRC cells at indicated time points after actinomycin D treatment (Figure S8). These results were consistent with the knowledge that YTHDF2 tended to facilitate targeted mRNA decay.^30^ The interaction between YTHDF2 protein and RAB5A mRNA was then verified by RIP and RNA pull down assays (Figures 4I and S9). In addition, although YTHDF2 expression would not be affected by ALKBH5, the knockdown of YTHDF2 could counteract the degradation of RAB5A caused by ALKBH5 loss (Figure 4J). Collectively, overexpressed‐ALKBH5 could enhance the stability of RAB5A mRNA via an m6A‐YTHDF2‐dependent pathway.

### 3.5 RAB5A serves as an oncogene in CRC

**FIGURE 5:**
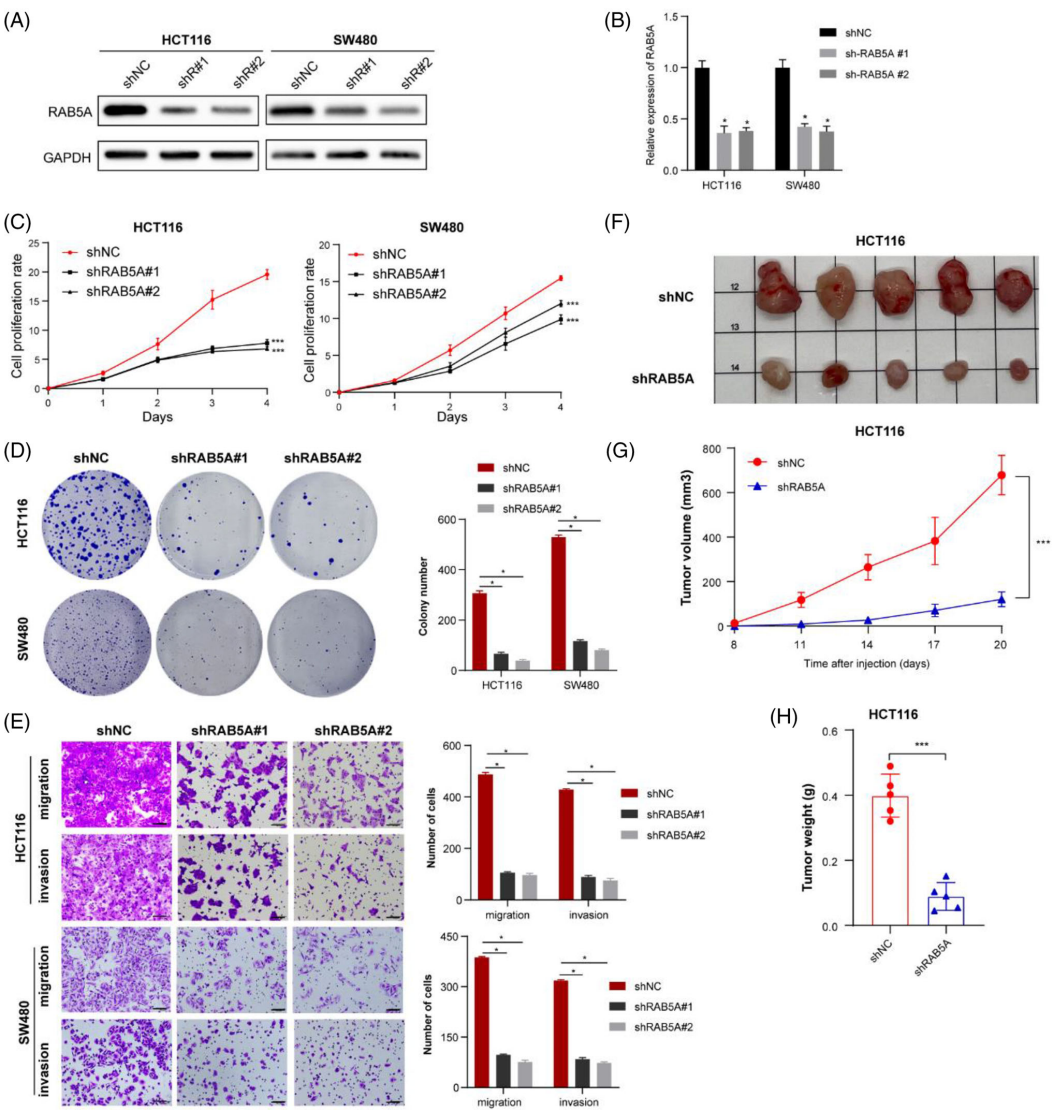
Knockdown of RAB5A inhibits the growth and motility of CRC cells. The efficiency of knockdown of RAB5A in HCT116 and SW480 cell lines was validated by western blotting (A) and qPCR (B). (C, D) CCK‐8 (C) and colony formation (D) assays were applied to evaluate the proliferation ability of HCT116 and SW480 cells with knockdown of RAB5A. (E) Transwell assays of HCT116 and SW480 cells were applied to measure their migration and invasion abilities (scale bars, 50 µm). (F–H) Representative subcutaneous xenograft tumour image (F) from sacrificed mice and their tumour volume curves (scale plate: 10 mm × 10 mm for each square), (G) and tumour weight (H) were shown with models injected with stable RAB5A‐knockdown HCT116 cells (*n* = 5).

To gain insight into the phenotype of RAB5A in CRC, the expression of RAB5A in HCT116 and SW480 cells was silenced using shRNAs targeting RAB5A (shRAB5A), and the transfection efficiency was also validated by WB and qPCR (Figure 5A and B). CCK‐8 assays and colony formation revealed that the silence of ALKBH5 strongly inhibited the growth and viability of CRC (Figure 5C and D). As shown in the Transwell assays, loss‐of‐function of RAB5A markedly impaired the migrative and invasive abilities of CRC cells (Figure 5E). In vivo experiments, we also found that silencing RAB5A expression could drastically diminish tumour weights and volumes of xenografted tumours compared to control group (Figure 5F‐H). In summary, RAB5A was activated during CRC tumourigenicity and promotes the oncogenesis of CRC.

### 3.6 ALKBH5 promotes CRC progression through RAB5A‐mediated regulation

**FIGURE 6:**
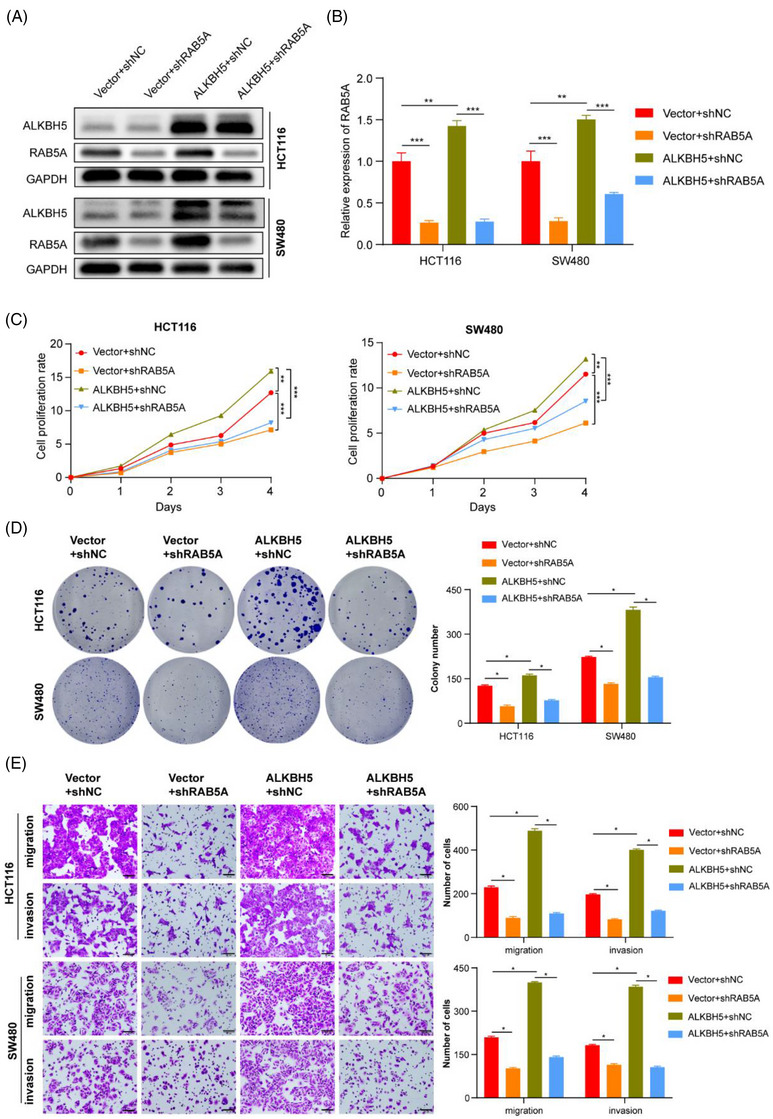
Knockdown of RAB5A reverses the malignant effects of ALKBH5 in vitro. A‐B Western blotting (A) qPCR (B) were performed to examine RAB5A expression in different transfected cell groups. (C–E) Rescue experiments by CCK‐8 (C), colony formation (D) and Transwell assays (E) were conducted to evaluate the effect of RAB5A interference on the of growth and motility of HCT116 and SW480 cells with overexpressed ALKBH5.

To prove that the above phenotypes induced by ALKBH5 were also regulated by the ALKBH5‐RAB5A axis, we then designed some rescue functional assays in vitro. WB and qPCR assays were conducted to validate that the overexpression of RAB5A mediated by overexpressing ALKBH5 was strikingly reduced by RAB5A knockdown in HCT116 and SW480 cells (Figure 6A and B). Silencing of RAB5A would extensively reverse the cell proliferation, colony formation, migration and invasion promoted by overexpression of ALKBH5 in cells (Figure 6C‐E).

## 4 DISCUSSION

Accumulating numbers of studies continue to validate that m6A RNA modification exerts crucial roles in pathological and physiological conditions, especially in the development of multiple cancers including CRC. Among them, the m6A regulatory proteins (‘writers’, ‘erasers’ and ‘readers’) act as key roles in the tumour‐promoting and anti‐tumour effects. As the central component of the N6‐methyltransferase complex, METTL3 and METTL14 showed the opposite effect in CRC proliferation and metastasis via diverse mechanisms.^10^, ^11^, ^12^ For m6A erasers, two studies reported ambiguous results about FTO in CRC. Ruan et al. found that FTO exerted a tumour‐suppressive role by inhibiting MTA1 expression in an m6A‐IGF2BP2‐dependent manner,^31^ while Wang et al. implied that FTO could promote CRC progression and chemotherapy resistance via demethylating G6PD/PARP1.^32^ These studies suggested the complicated biological function of m6A in CRC. However, the role of ALKBH5 participated in the initiation and progression of CRC remains controversial. Guo et al. found that ALKBH5 facilitated the malignant behaviour of CRC via modulating lncRNA NEAT1 demethylation.^33^ While a most recent study identified that ALKBH5 could suppress CRC progression by decreasing PHF20 mRNA stability via an m6A‐dependent pathway.^34^ These controversial results reflected that the consequences of aberrant m6A deposition may depend on the cancer heterogeneity, and even in the same tumour, it may depend on specific cell types or dysregulation of the downstream target genes and signalling pathways. In our research, we screened the expression of ALKBH5 in CRC from the cellular to tissue levels and further to clinical relevance using a large in‐house cohort with sufficient patients. We found that ALKBH5 was overexpressed in CRC and independently associated with worse prognosis for CRC patients. Furthermore, ALKBH5 was identified to present with oncogenic phenotypes in two CRC cell lines different from the above studies. Therefore, the downstream target of ALKBH5 merited further investigation.

As a second discovered m6A demethylase, ALKBH5 has been shown to regulate mRNA metabolism by decreasing m6A levels in nuclear specks. In the field of cancer research, ALKBH5 was reported to enhance self‐renewal and proliferative ability of cancer stem cells (CSC) in glioblastoma.^17^ and mediate hypoxia‐induced phenotypes of breast cancer stem cells.^35^ It also promoted the invasion and metastasis of gastric cancer,^36^ ovarian cancer^37^ and KRAS‐mutated lung cancer.^38^ In contrast, ALKBH5 was mostly reported to play tumour‐suppressive roles and inhibit cancer malignancy of hepatocellular carcinoma,^28^ osteosarcoma,^18^ esophageal cancer^39^ and pancreatic cancer.^14^, ^15^ In addition, previous studies unveiled a more important function of ALKBH5 in the tumour immune microenvironment. ALKBH5 could promote the expression of PD‐L1 in intrahepatic cholangiocarcinoma^16^ and the deletion of ALKBH5 would sensitise melanoma and CRC to immunotherapy,^40^ which indicated that ALKBH5 might be a potential biomarker for immune checkpoint blockade therapy in multiple cancers. With systematic analysis of high‐throughput sequencing and low‐throughput validation, we revealed a novel m6A methylation‐involved mechanism featured by ALKBH5‐RAB5A axis in CRC. The present study demonstrated that ALKBH5 exerted an oncogenic role in CRC by downstream regulating RAB5A mRNA methylation. Mechanistically, we mainly elucidated that the low RAB5A mRNA level by ALKBH5 knockdown was due to the accelerated decay of RAB5A mRNA depending on m6A reader protein YTHDF2, decreasing the RAB5A protein level as a result, and RAB5A, a member of RAS oncogene family, was subsequently identified as a key oncogenic driver in CRC.

Mutations and aberrant expressions of small GTPases of the Ras superfamily (e.g. KRAS) are acknowledged among the key driving factors for tumour genesis and progression.^41^, ^42^ However, whether m6A modulators contribute to regulating the expression levels of small GTPase proteins remains to be investigated. Recently, Yang et al. conducted a comprehensive analysis based on the high‐throughput quantitative proteomic approach to clarify the alterations in small GTPases regulated by m6A regulatory proteins.^43^ They identified for the first time that there indeed existed a subset of small GTPases, such as Rab GTPases, which was modulated by ALKBH5 and METTL3. Therefore, in the future, studies to find out the specific m6A‐mediated GTPases‐related genes or pathways constituted major mechanisms would provide novel insights for cancer research. Intriguingly, the present study has systematically illustrated the cancer‐related behaviour of RAB5A, a member of the Rab GTPases subfamily, and its upstream relation with the m6A‐modulator ALKBH5. RAB5A was best known as a master regulator of intracellular vesicle transport and could induce cancer cells invasion and metastasis.^44^ In clinic, elevated expression and activity of RAB5A was correlated with metastasis in breast^45^ and lung^46^ cancers. Currently, there were few studies focusing on the function of RAB5A in CRC. Wang et al.^47^ reported that RAB5A activation would promote colorectal tumourigenesis via the EGFR/MEK/ERK signal pathway. Similarly, Takeda et al.^48^ demonstrated that RAB5A played a key role in the maintenance of colorectal CSC survival via regulation of the mitophagic pathway. In this study, we found that RAB5A knockdown significantly suppressed the proliferation, migration and invasion of CRC cells in vitro and inhibited subcutaneous tumour growth in vivo. Moreover, we also found that the disruption of RAB5A could efficiently eliminate the carcinogenic phenotypes of ALKBH5. Although diverse mechanisms contributed to the progression of CRC, targeting ALKBH5‐RAB5A axis may be part of a potential treatment approach to foster a therapeutic response against CRC. It was noteworthy that mefloquine hydrochloride, an antimalarial drug, was identified as a novel RAB5A inhibitor and could efficiently disrupt the tumourigenic activity of colorectal cancer stem cells.^48^ Hence, we speculated that mefloquine combined with other chemotherapeutic agents might be promising therapeutic regimens via targeting the ALKBH5‐RAB5A signal axis and could better improve the prognosis of CRC.

## 5 CONCLUSION

**FIGURE 7:**
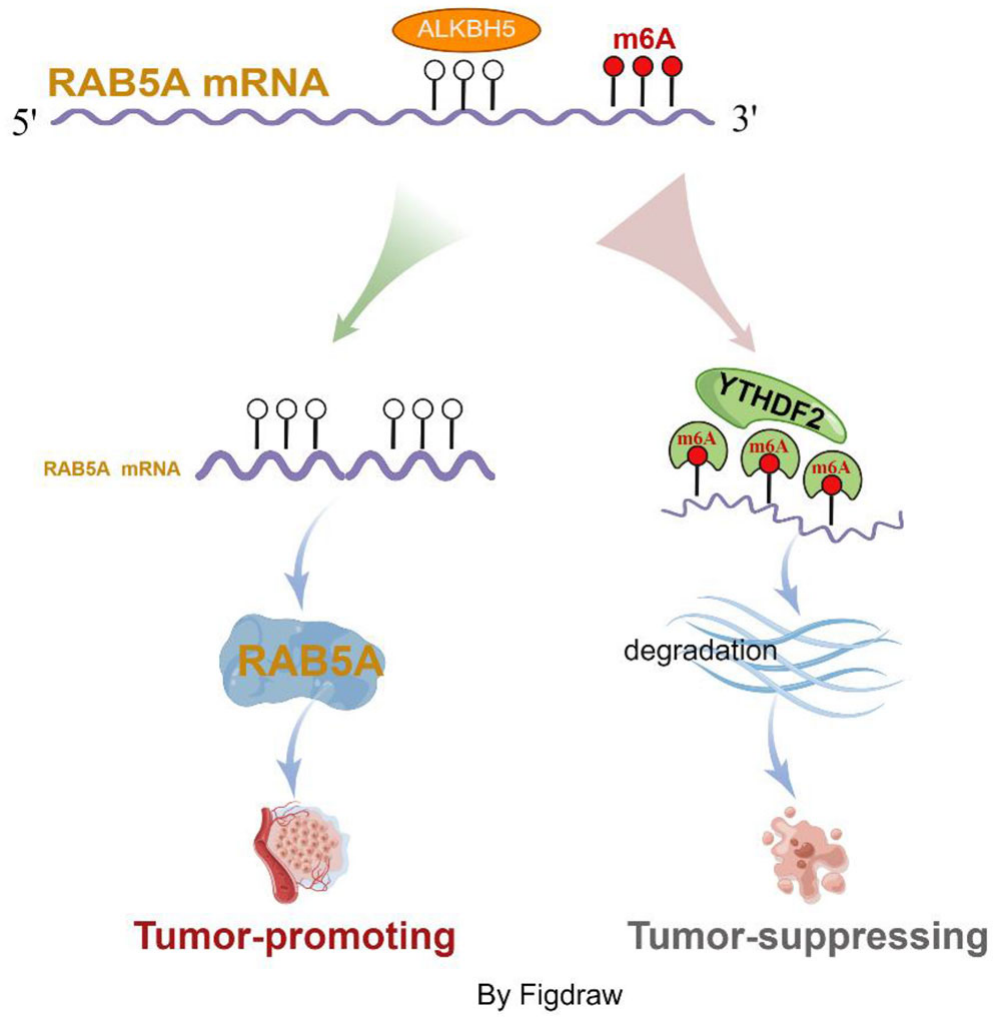
A schematic illustration was proposed to summarise our findings about ALKHB5‐guided m6A modulation on RAB5A. In brief, ALKBH5‐deficiency enhanced the m6A methylation on the 3′UTR region of RAB5A mRNA and subsequently accelerated degradation of RAB5A mRNA in a YTHDF2‐dependent manner, thereby inactivated the RAB5A protein level and suppressed the tumourigenesis of CRC.

In summary, this study validated that the higher expression of ALKBH5 was closely correlated with adverse clinicopathological factors and poor survival outcomes in CRC. Functionally, ALKBH5 facilitated the proliferation and motility of CRC cells. Moreover, we revealed that RAB5A was the direct target of ALKBH5 and served as a novel oncoprotein in CRC. Mechanically, ALKBH5 attenuated the m6A methylation in the 3′UTR region of RAB5A mRNA and subsequently prevented the YTHDF2‐mediated degradation of RAB5A mRNA (Figure 7). Thus, ALKBH5‐RAB5A axis was closely participated in the tumourigenicity of CRC. Taking together, our results extend a novel molecular mechanistic understanding of ALKBH5 in CRC oncogenesis and provide more new insights into developing effective treatment strategies for CRC.

## FUNDING

This work was supported by the National Natural Science Foundation of China (No. 81972245, YL; No. 82173067, YL; No. 81902877, HY; No. 82272965, HY; No. 32100627, YZ), the Natural Science Foundation of Guangdong Province (No. 2022A1515012656, HY; No. 2021A1515010134, MH; No. 2020A1515010036, XL), the Project 5010 of Clinical Medical Research of Sun Yat‐sen University‐5010 Cultivation Foundation (No. 2018026, YL), the Science and Technology Program of Guangzhou (202201011004, HY), the Talent Project of the Sixth Affiliated Hospital of Sun Yat‐sen University (No. P20150227202010251, YL), the Excellent Talent Training Project of the Sixth Affiliated Hospital of Sun Yat‐sen University (No. R2021217202512965, YL), the Sixth Affiliated Hospital of Sun Yat‐sen University Clinical Research‐‘1010’ Program (MH; YL), the Program of Introducing Talents of Discipline to Universities, and National Key Clinical Discipline (2012), Fundamental Research Funds for the Central Universities, Sun Yat‐sen University (23ykbj007).

## CONFLICT OF INTEREST STATEMENT

The authors declare that they have no competing interests.

## Supporting information

Supporting Information

Supporting Information
