# Supplementary material for: RNA demethylase ALKBH5 promotes colorectal cancer progression by posttranscriptional activation of RAB5A in an m6A‐YTHDF2‐dependent manner
Source: Clin Transl Med. 2023 May 18;13(5):e1279. doi: 10.1002/ctm2.1279 (PMC10196219; doi:10.1002/ctm2.1279)
Supplement: Supplementary file 1 — Supporting Information [file CTM2-13-e1279-s001.docx]

**Supplementary Tables**

**Table S1.** The sequence of plasmids or siRNAs.

| **Name** | **Oligonucleotides (5’-3’)** |
| --- | --- |
| **OE-ALKBH5** | Forward sequence: ATGGCGGCCGCCAGCGGC |
|  | Reverse sequence: TCAGTGCCGCCGCATCTT |
|  | **Target sequences** |
| **shALKBH5#1** | GCTGCAAGTTCCAGTTCAAGC |
| **shALKBH5#2** | GCTTCAGCTCTGAGAACTACT |
| **shRAB5A#1** | CAAGGCCGACCTAGCAAATAA |
| **shRABA#2** | GAATTTCAAGAGAGTACCATT |
| **siYTHDF1#1** | GATACAGTTCATGACAATGA |
| **siYTHDF1#2** | GAAACGTCCAGCCTAATTCT |
| **siYTHDF2#1** | CTGGATATAGTAGCAATTA |
| **siYTHDF2#2** | CCATTACTAGTAACATCGT |
| **siYTHDF3#1** | GGACGTGTGTTTATAATTA |
| **siYTHDF3#2** | GACTAGCATTGCAACCAAT |

**Table S2.** Sequences of primers utilized in this study.

| **Primer names** | **Sequences (5’-3’)** | |
| --- | --- | --- |
|  | **Forward** | **Reverse** |
| **ALKBH5** | CGGCGAAGGCTACACTTACG | CCACCAGCTTTTGGATCACCA |
| **RAB5A** | AGACCCAACGGGCCAAATAC | GCCCCAATGGTACTCTCTTGAA |
| **DNAJB1** | AAGGCATGGACATTGATGACC | GGCCAAAGTTCACGTTGGT |
| **CCSER2** | AGTGCCTCTACGGTCAAGTAT | ACCCCAAATGTTCATTCCCATTG |
| **DYNLRB1** | GAAGGGAGTGCAGGGAATCAT | ATGGTGCTCTTGATGGGAATG |
| **PLCD3** | CCACAACACCTATCTGACTGAC | CTGGGCAAAGGCCCTAACAT |
| **HIP1R** | CGAGCAGTTCGACAAGACCC | GTGTGCCCAGAATGATGCG |
| **YTHDF1** | ACCTGTCCAGCTATTACCCG | TGGTGAGGTATGGAATCGGAG |
| **YTHDF2** | AGCCCCACTTCCTACCAGATG | TGAGAACTGTTATTTCCCCATGC |
| **YTHDF3** | TCAGAGTAACAGCTATCCACCA | GGTTGTCAGATATGGCATAGGCT |
| **RAB5A-MeRIP** | TGCATGGGTCCCTCTCACTA | TCCCCCTAACCTTCCAATGA |

**Table S3.** The sequences of the wild-type and m6A sites mutated RAB5A.

| **Name** | **Sequences of region** |
| --- | --- |
| **RAB5A 3’UTR-WT** | GCTAGCTGAGCTCCAACCTTTTGCCTAAGGCATAAGCAATAGAAGCAGCAGTTAGCTCATTGATAATTCTCAGTACATTGAGATAAGCAATAGTTCCAGCATCTTTGGTAGCTTTGATGCTGAGAATCATTAAACCAAGCTGATATTGTGACCACAGCATTGGTAACACTCTTTCCAAGGTAGGCTTCTGCCATTTCCTTCATCTTTGTCAGAATAACAGAGCAGCTGTCCCCAACCTTTTTGGCACCAGGGACCAGTTTCATGGAAGACAATTTTTCCACAGACCAAGTTGCAGGGGGTAGGATCGGGGGAAGATAGTTTTAGGACAATTCAAGCACATTACATTTATTGTGCACTTTATATTATTATTACATTGTAATATATAATGAAATAATTATATAACTCACCATAATGTACAATCAGTAGGCTCGAG |
| **RAB5A 3’UTR-Mut** | GCTAGCTGAGCTCCAACCTTTTGCCTAAGGCATAAGCAATAGAAGCAGCAGTTAGCTCATTGATAATTCTCAGTACATTGAGATAAGCAATAGTTCCAGCATCTTTGGTAGCTTTGATGCTGAGAATCATTAACCCAAGCTGATATTGTGCCCACAGCATTGGTAACACTCTTTCCAAGGTAGGCTTCTGCCATTTCCTTCATCTTTGTCAGAATACCAGAGCAGCTGTCCCCAACCTTTTTGGCACCAGGGCCCAGTTTCATGGAAGCCAATTTTTCCACAGCCCAAGTTGCAGGGGGTAGGATCGGGGGAAGATAGTTTTAGGCCAATTCAAGCACATTACATTTATTGTGCACTTTATATTATTATTACATTGTAATATATAATGAAATAATTATATACCTCACCATAATGTACAATCAGTAGGCTCGAG |

**Supplementary Figures**


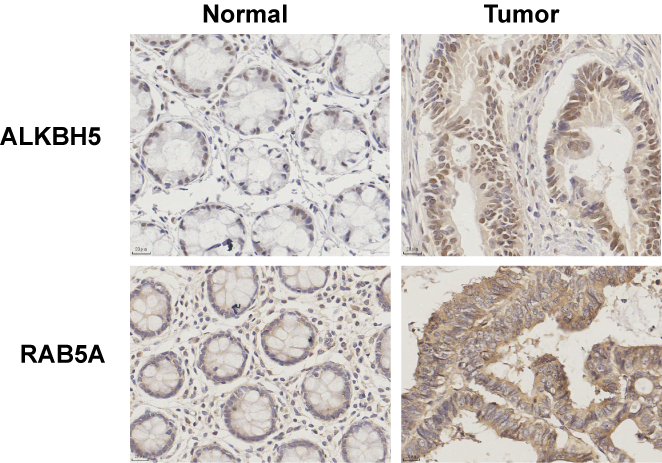


**Figure S1.** The representative images of IHC staining of ALKBH5 and RAB5A expression in paired CRC tissues and adjacent normal tissues.


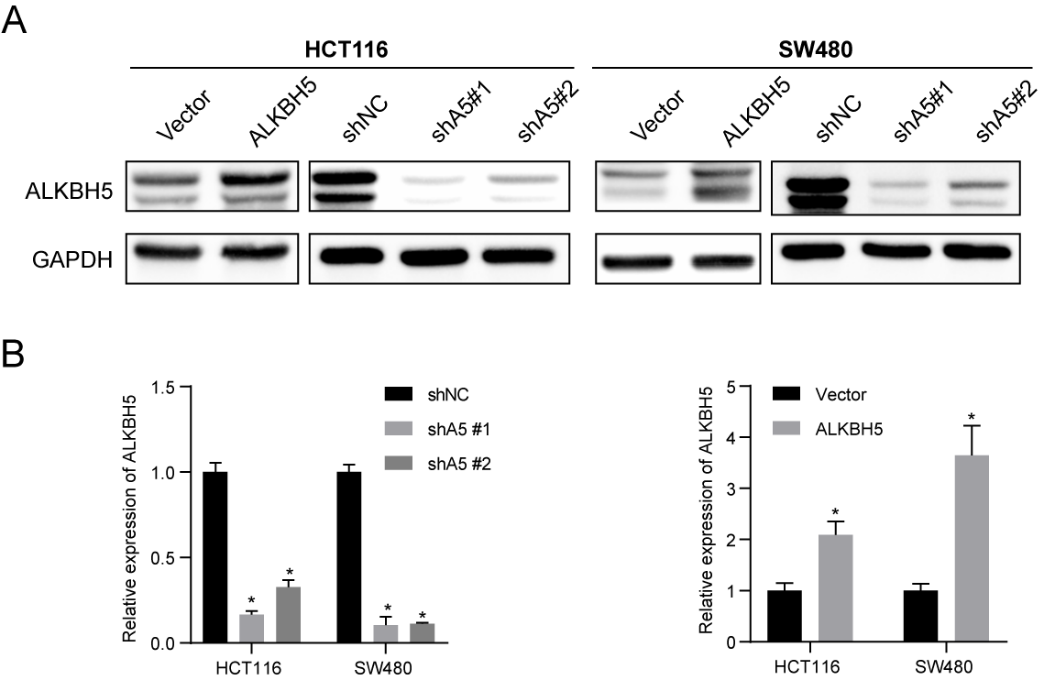


**Figure S2.** The efficiency of overexpression or knockdown of ALKBH5 in HCT116 and SW480 cell lines was validated by western blotting (**A**) and qPCR (**B**).


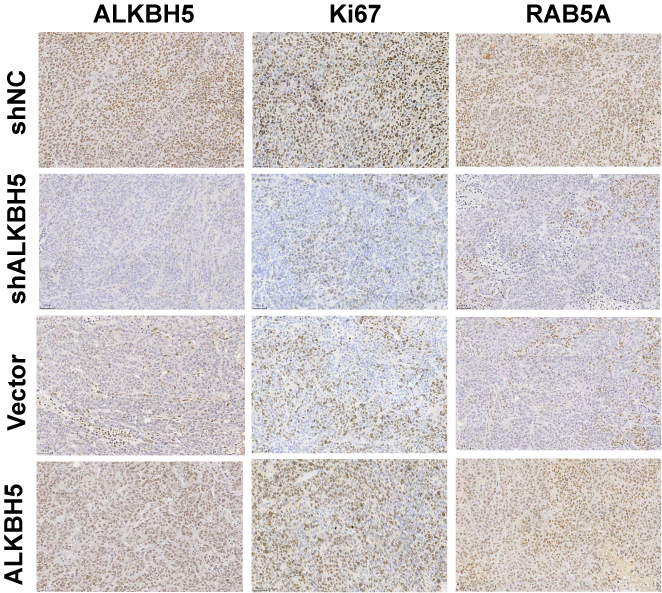


**Figure S3.** Typical IHC images of subcutaneous tumors using ALKBH5-overexpressed or -silenced transfected cells and their control groups were shown. Staining of ALKBH5 was applied to validate the transfection efficiency, while the intensity of Ki67 staining represented the proliferation capability of tumors and the staining of RAB5A represented the correlation to ALKBH5 expression.


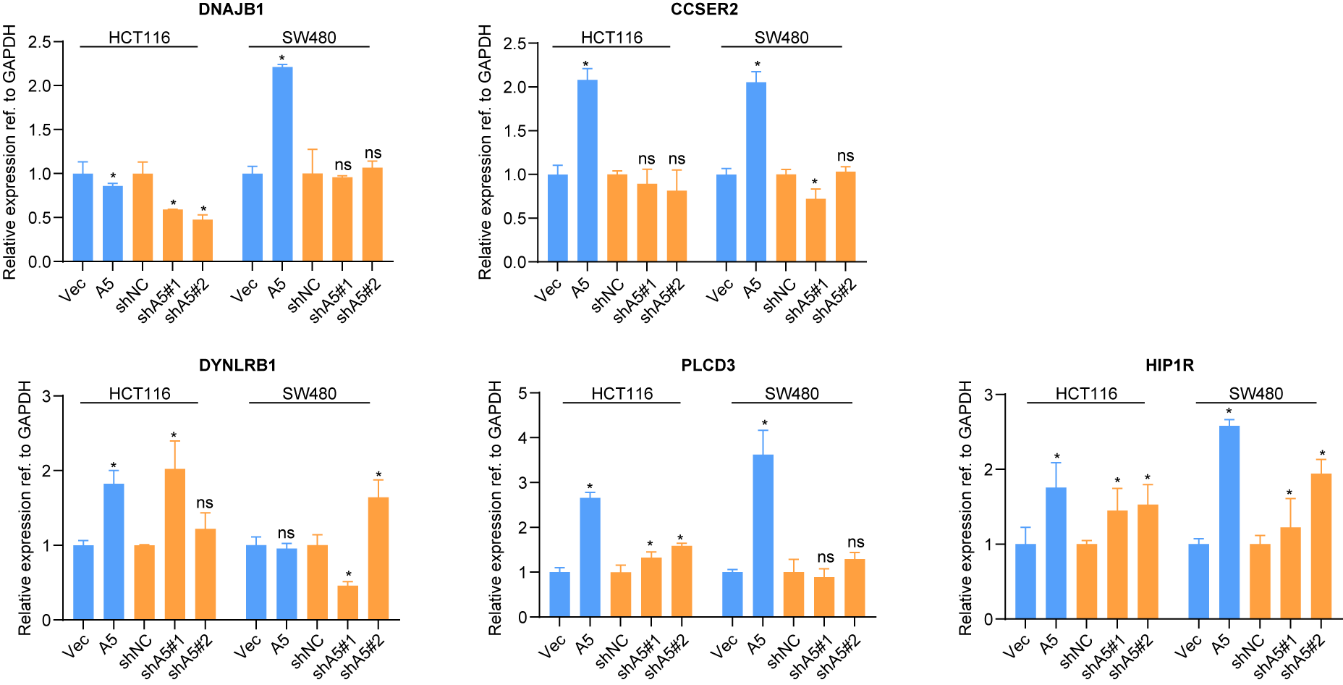


**Figure S4.** RNA levels of DNAJB1, CCSER2, DYNLRB1, PLCD3, and HIP1R were examined in ALKBH5-silenced or -overexpressing cells, respectively.


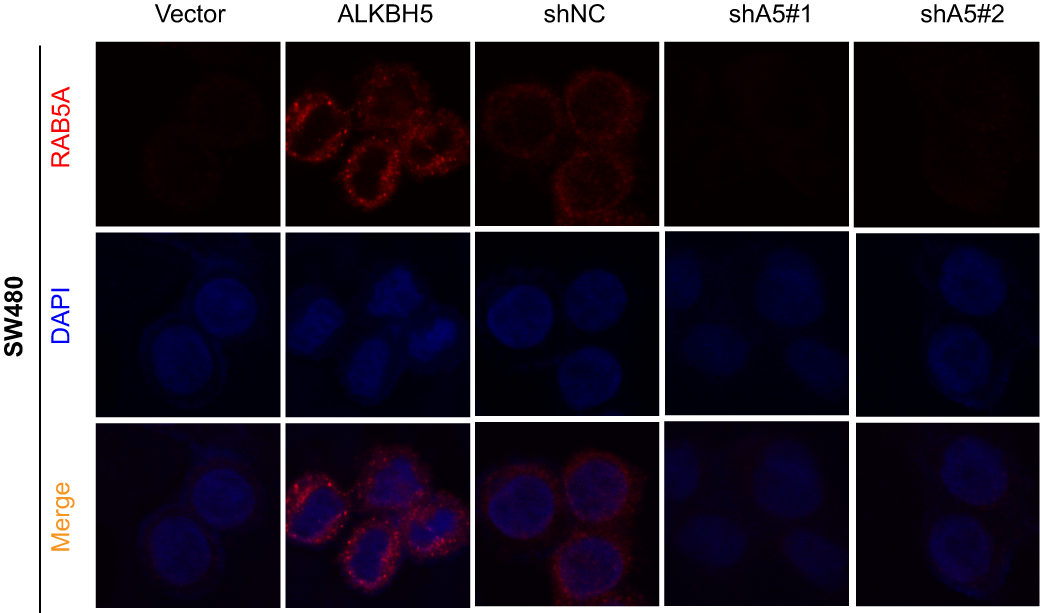


**Figure S5.** Alterations of RAB5A protein represented with immunofluorescent imaging were detected in ALKBH5-silenced or -overexpressing SW480 cells.


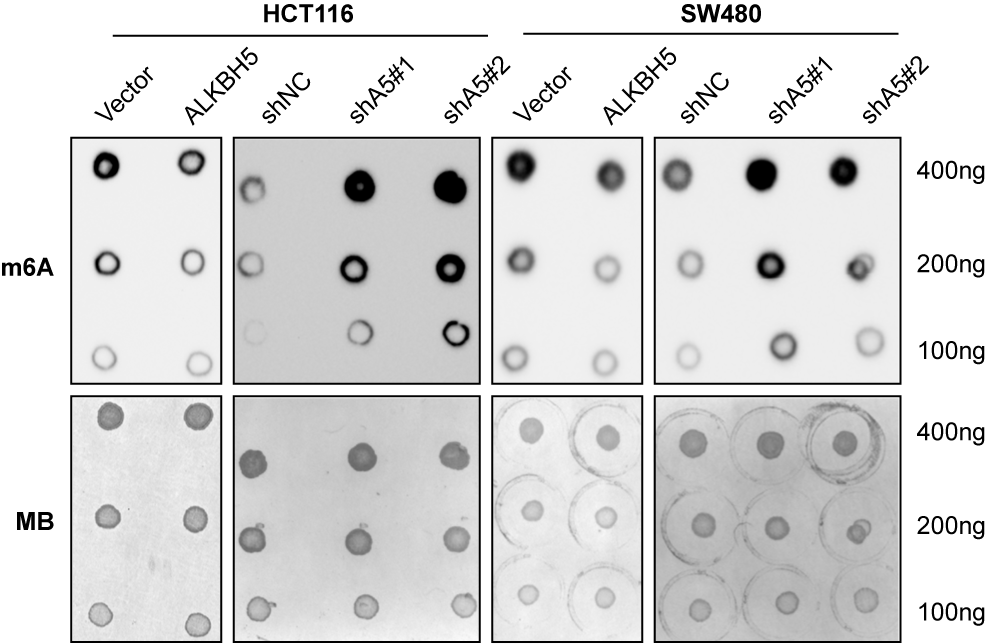


**Figure S6.** The m6A dot blot assay of HCT116 and SW480 cells transfected with knockdown and overexpression of ALKBH5 or its corresponding control lentivirus. The gray background above was methylene blue staining (MB, loading control).


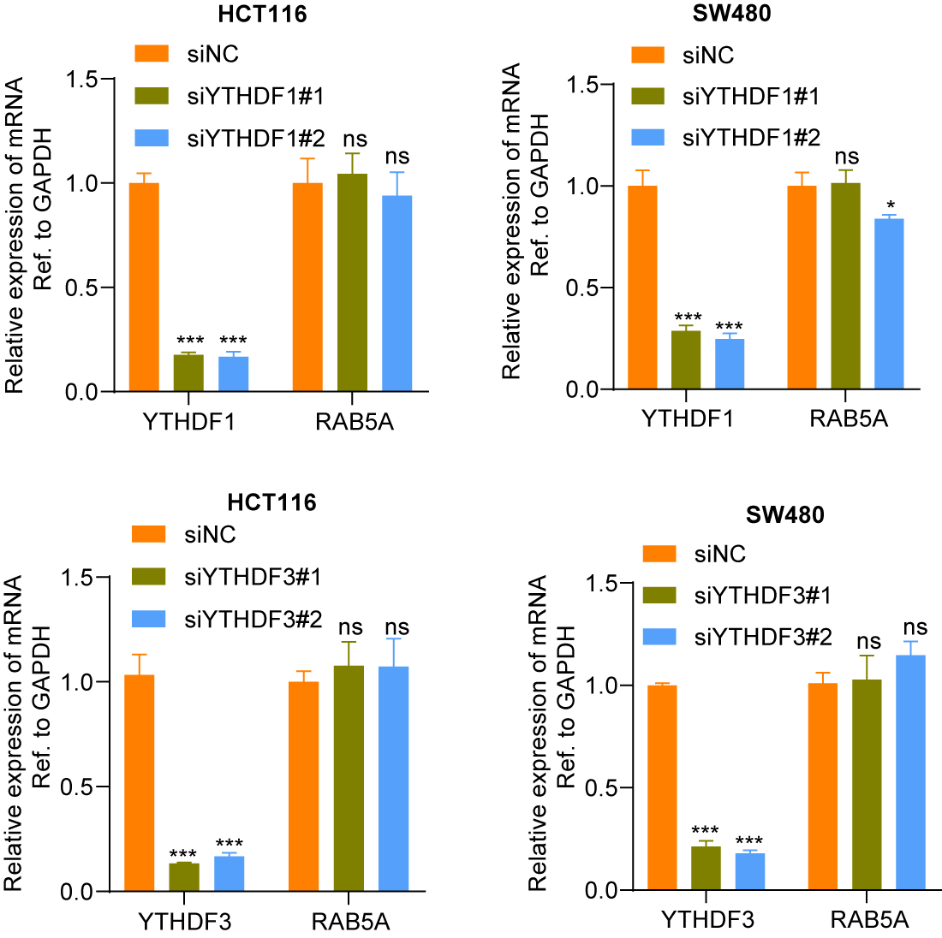


**Figure S7.** YTHDF1/3 were knockdown in two CRC cells followed by the measurement of RAB5A expression via qPCR.


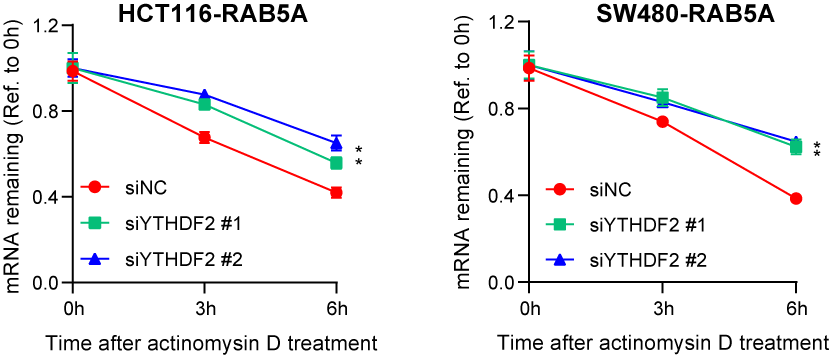


**Figure S8.** YTHDF2-silenced cells were treated with actinomycin D at the indicated time points. RNA decay rate was determined to estimate the stability of RAB5A (normalized to the expression at 0 h).


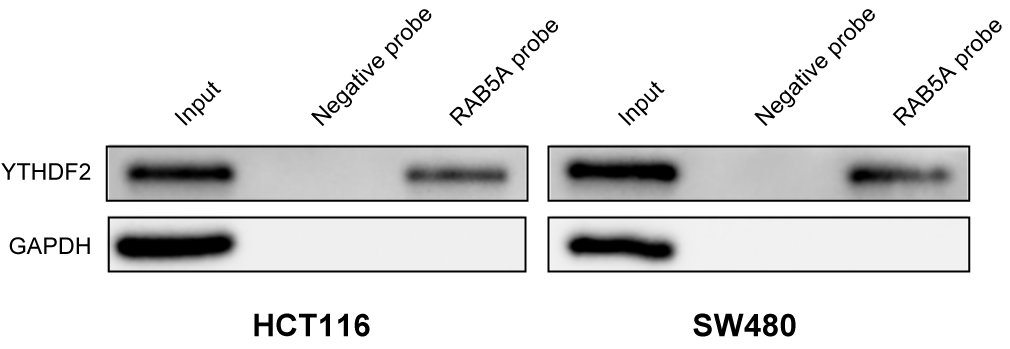


**Figure S9.** RNA pull-down assay was used to verify the binding of YTHDF2 to RAB5A mRNA. RNA pull-down was carried out using an RNA pull-down kit (BersinBio, China) following its recommended protocols. The proteins were pulled down by incubating the cell lysates with biotin-labeled RAB5A probes or Negative control probes and streptavidin beads. The retrieved protein was eluted and then separated and identified using western blot.
